# Comparison of depressive symptoms and eating behaviors among Japanese university students with subthreshold depression before and during the COVID-19 pandemic

**DOI:** 10.3389/fpsyt.2025.1480992

**Published:** 2025-06-16

**Authors:** Yoshie Miyake, Koki Takagaki, Atsuo Yoshino, Yuri Okamoto

**Affiliations:** Health Service Center, Hiroshima University, Higashi-Hiroshima, Japan

**Keywords:** COVID-19, eating behavior, stress coping, subthreshold depression, university student

## Abstract

**Background:**

The COVID-19 pandemic had exposed university students to unusual environmental stresses. High stress levels have been reported among adolescents with depressive symptoms. Subthreshold depression is highly prevalent among university students who are at high risk of developing clinical depression and other psychiatric disorders such as eating disorders. Therefore, this study aimed to investigate the effects of the pandemic on depressive symptoms and eating behaviors among Japanese university students with subthreshold depression from a cohort study.

**Methods:**

A total of 555 fourth-year university students, 261 students before the pandemic and 294 students during the pandemic, were participated. The inclusion criteria were age 18–19 years and subthreshold depression upon entering the university and completed questionnaires at both university entrance and during their fourth year. We examined differences in depressive symptoms (Beck Depression Inventory II), eating behaviors (Eating Attitudes Test-26 and Bulimic Inventory Test, Edinburgh [BITE]), stress coping (Coping Inventory for Stressful Situations [CISS]), and the frequencies of the three depression groups (clinical, subthreshold, and healthy) before and during the pandemic. We also investigated the relationship between changes in depression and stress coping during the pandemic.

**Results:**

There was no significant differences in depression frequency in the fourth year before and during the pandemic. However, the two-way ANOVA on the grade and time revealed significant interaction effects on the BITE scores for females (*p* < 0.05), and the BITE scores were significantly higher for fourth-year female students during the pandemic than those when they entered the university. Although a decrease in the CISS-emotion-oriented score was associated with a reduction in depressive symptoms both before and during the pandemic, a decrease in the CISS-task-oriented score was associated with an increased risk of depressive symptoms during the pandemic only in males.

**Conclusion:**

Our results suggest that not only depressive but also bulimic symptoms should have been monitored among university students with subthreshold depression during the pandemic. Attention to stress-coping behaviors may be important for interventions to prevent the deterioration from subthreshold depression to clinical depression during a pandemic. However, the results could have been affected by many other factors, and further research is required.

## Introduction

The coronavirus disease 2019 (COVID-19) pandemic has negatively affected adolescents’ mental health. Adolescents may have been more susceptible to the mental health impacts of the public health crisis than adults (1). Many studies have reported that the rates of depressive symptoms in university students during the pandemic were comparatively higher than before the pandemic (2–5). University students have experienced very high levels of anxiety and stress during the pandemic (6, 7). Studies of Japanese youth have demonstrated high rates of depressive symptoms (7, 8). Suicide rates and the number of high-risk students with suicidal ideation in Japanese universities increased during the pandemic (8, 9). Japan has not experienced any serious damage from new infectious diseases and the history of escapes might have given the Japanese greater anxiety and stress about new infectious diseases (10). Previous studies have reported that the pandemic exerted a negative influence on people who were already suffering from mental disorders (11, 12). Subthreshold depression is highly prevalent in adolescents, and is defined as clinically significant depressive symptoms that do not meet the diagnostic criteria for clinical depression (13). Despite being less severe than clinical depression, subthreshold depression in adolescence is characterized by high levels of comorbidities, functional impairment, and suicidal thoughts and behaviors (14). People with subthreshold depression are approximately twice as likely to develop clinical depression as those without (15). Therefore, investigating the effects of the pandemic on depressive symptoms of university students with subthreshold depression is crucial for the prevention and early intervention. The estimated prevalence of subthreshold depression is higher than that of clinical depression (14). A previous study suggested that subthreshold depression impairs young people’s quality of life and places them at a greater risk for developing clinical depression (16, 17). During the pandemic, high stress levels have been reported in individuals with pre-existing mental health disorders, especially anxiety and depression (18). Although information about the pandemic effects on the mental health of university students with subthreshold depression is essential, few studies have investigated them. A study showed that the prevalence of subthreshold depressive episodes were 14.3% and associations with social and economic factors differ according to the level of such symptoms in university students during the pandemic (19). People with subthreshold depression are more likely to develop other psychiatric disorders such as eating disorders and stress-related diseases (14, 16, 17, 20). A study of university students found an overall low quality of life during the pandemic (21). Pandemic-induced disruptions in daily routines caused changes in usual patterns of eating, physical activity and stress coping behavior (22–24). Therefore, further research comparing the mental health, including depressive symptoms and eating behaviors, of university students with subthreshold depression before and during the pandemic is needed. Moreover, previous studies have suggested that stress coping style was one factor contributing to adolescents’ increased vulnerability to depression (25). Stress coping behaviors play an important role in reducing stress and consequently affect the occurrence of stress−related diseases, such as depression (26, 27). Therefore, understanding the role of stress-coping behaviors in the development of depression during a pandemic would provide a useful perspective for providing more effective support to university students with subthreshold depression during unusual times.

Several studies have reported that pandemic worsened the eating disorder psychopathology (28, 29). University students are particularly affected by mood and eating problems (30). Eating disorders are more common among females than males and are an important cause of physical and psychosocial morbidity in young females. An increase in eating disorders is associated with academic stress, interpersonal relationships and environmental factors. The pandemic has been a leading cause of stress and feelings of loss of control, both of which are related to the onset and progression of eating disorders (22). Depressive symptoms, including subthreshold levels, are associated with an increased risk of disordered eating behaviors (14, 20, 31, 32). However, little is known about the effects of the pandemic on eating behaviors among university students with subthreshold depression.

This study investigated the effects of the COVID-19 pandemic on depressive symptoms and eating behaviors among Japanese university students with subthreshold depression upon university entrance, deepening the analysis of the relationship between changes in depressive symptoms and stress coping behaviors. In this study, we evaluated depressive symptoms, eating behaviors, and stress coping styles upon university entrance and during the fourth year, and compared them between students before and during the pandemic. We hypothesized that fourth-year students would display more depressive symptoms and disordered eating behaviors during rather than before the pandemic.

## Methods

### Participants

The participants were Japanese fourth-year students at Hiroshima University from 2017–2019 (before the pandemic) and 2021–2022 (during the pandemic). The inclusion criteria were age 18–19 years at the time of entry into university, completed questionnaires at both university entrance and during their fourth year, and had subthreshold depressive symptoms upon university entrance.

### Procedures

This study was conducted at two health checkup time points: university entrance and the first quarter of the fourth year. Questionnaires were administered as part of annual checkups. Students were informed and guided to fill out the questionnaire through mail (first-year students) or bulletin board (fourth-year students), and they answered the questionnaire. The questionnaire consisted of two sections. The first section recorded participant baseline (age, sex, height, and weight). The second section included four scales: Beck Depression Inventory II (BDI-II) (33), Eating Attitudes Test-26 (EAT-26) (34), Bulimic Inventory Test, Edinburgh (BITE) (35), and Coping Inventory for Stressful Situations (CISS) (36). Based on previous studies, we divided the students into three categories based on depressive symptoms determined by BDI-II scores for clinical, subthreshold, and healthy groups at university entrance (31, 37). Therefore, students were classified as part of the subthreshold group according to BDI-II scores ranging from 10 to 17 at university entrance.

To investigate the pandemic effects on the mental conditions of fourth-year students, we examined BDI-II, EAT-26, BITE and CISS scores of the fourth-year students who had subthreshold depressive symptoms upon university entrance, and then compared the scores before and during the pandemic. We also examined the correlation between the BDI-II and the EAT-26 and BITE and defined fourth-year students with severely disordered eating behaviors as having scores of 20 or more points on the EAT-26 and BITE. Moreover, we examined the differences in stress-coping abilities among students who deteriorated from subthreshold to clinical (exacerbated group), those who remained subthreshold (unchanged group), and those who improved from subthreshold to healthy (improved group). The study protocol was reviewed and approved by the Ethics Committee of Hiroshima University School of Medicine, Japan (approval number: E2019-1767) and was conducted in accordance with the Declaration of Helsinki. Although the questionnaires were administered as part of an annual checkup, informed consent was obtained in the form of opt-outs.

### Measures

#### Beck depression inventory II

The original BDI-II, developed by Beck et al. (33), consists of 21 self-reported items rated on a 4-point scale and is used to measure depressive symptoms. The cutoff point for clinical depression is a score of 18 on the BDI-II. A cutoff score of 18 yielded a sensitivity of 94% and a specificity of 92%, and a cutoff score of 10 yielded a sensitivity of 100% and a specificity of 70% (37). The clinical group was defined as having BDI-II scores of 18 or more, the subthreshold group was defined as having scores ranging from 10 to 17, and the healthy group was defined as having scores of 9 or less. Cronbach’s alpha coefficient was 0.87 (38). The reliability and validity of the Japanese version of the BDI-II have been previously demonstrated (39).

#### Eating attitudes test-26

The EAT-26 is a 26-item self-report questionnaire that assesses eating attitudes and is a reliable and valid instrument (34). Answers are provided on a 6-point scale ranging from “not at all” to “extremely.” The cutoff score is 20 points; scores greater than 20 indicating a high possibility of eating disorders. Mann et al. (40) reported that a threshold of 20 yielded a sensitivity of 88% and a specificity of 96%. Cronbach’s alpha coefficients ranged from 0.85–0.94 (41). The reliability and validity of the Japanese version of the EAT-26 have been demonstrated (42, 43).

#### Bulimic inventory test, Edinburgh

The BITE is a self-reported measure of bulimic symptoms that consists of a symptom evaluation scale (30 items) and severity scale (6 items) (35). The symptom evaluation scale is scored as yes or no, with a minimum score of 0 and a maximum score of 30. The cutoff score was 20 points; symptom subscale scores greater than 20 indicated the presence of binge eating behavior and a high possibility of bulimia nervosa. Cronbach’s alpha coefficient was 0.96. The reliability and validity of the Japanese version of the BITE have been demonstrated (44).

#### Coping inventory for stressful situations

The CISS is a 48-item self-report measure scored on a 5-point scale that consists of three subscales to evaluate coping behaviors: task-oriented (CISS-T) (directly managing a stressor to reduce distress), emotion-oriented (CISS-E) (coping with the emotions and feelings aroused by the stressor), and avoidance-oriented (CISS-A) (seeking distractions) coping (36, 45). We used the Japanese version (46). Cronbach’s alpha coefficients ranged from 0.75–0.89 (47).

### Data analysis

SPSS version-28 (IBM Corporation, Armonk, NY, USA) was used for the statistical analyses. The participants’ characteristics were averaged. We used two-way analysis of variance (ANOVA) to compare scores by grade and time. Chi-square and residual analyses were used to compare the frequencies of depressive symptoms among the three groups. To investigate the relationship between changes in depressive symptoms and stress-coping behaviors, we conducted a two-way ANOVA to compare the CISS scores by grade and depression group. The statistical significance level was set at *p* < 0.05.

## Results

### Participants

**Figure 1:**
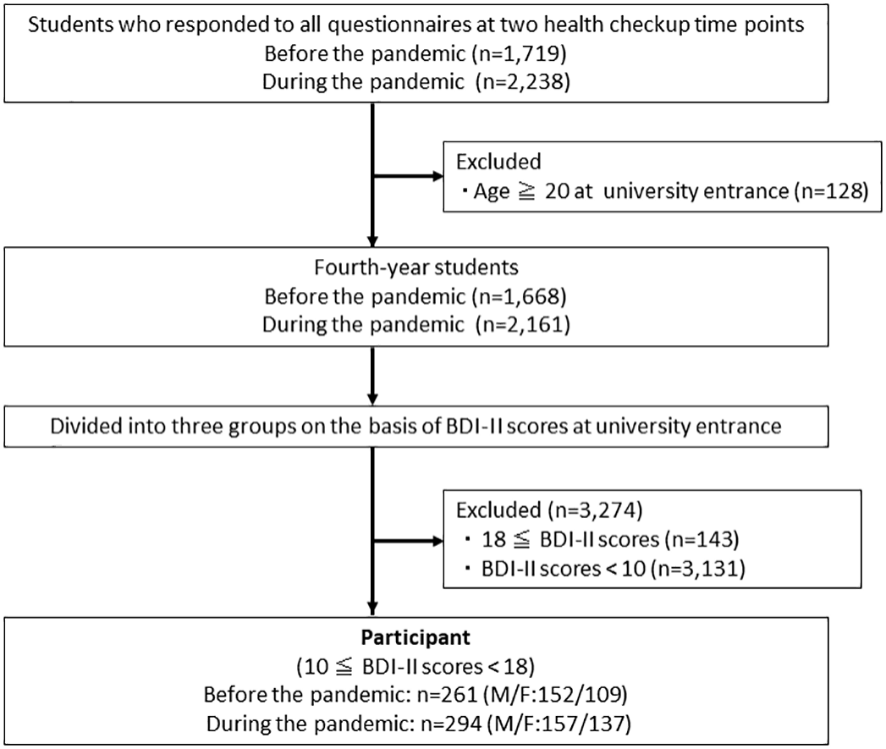
Participant flow chart.

A total of 555 students, who had subthreshold depressive symptoms at university entrance, completed questionnaires at both university entrance and during their fourth year participated. Of these 261 were fourth-year students (152 males and 109 females) before the pandemic and 294 were fourth-year students (157 males and 137 females) during the pandemic (**Figure 1**). The annual checkup results are presented in **Table 1**.

**Table 1:** Results of the annual checkups.

	Before the pandemic		During the pandemic	
	Mean (SD)		Mean (SD)	
	First-year	Fourth-year	First-year	Fourth-year
Male	n = 152		n = 157	
Age (years)	18.5 (0.5)	21.5 (0.5)	18.1 (0.3)	21.1 (0.3)
BMI (kg/m^2^)	21.0 (2.9)	21.3 (3.2)	21.0 (3.1)	21.0 (3.2)
Female	n = 109		n = 137	
Age (years)	18.4 (0.6)	21.4 (0.6)	18.2 (0.4)	21.2 (0.4)
BMI (kg/m^2^)	20.3 (2.7)	20.3 (2.5)	20.7 (2.5)	20.3 (2.4)

SD, standard deviation, BMI, Body Mass Index, n, number of students.

### Questionnaire results

Results of BDI-II, EAT-26, BITE and CISS are shown in **Table 2**. A score of 20 or more points on the EAT-26 and BITE indicates the presence of severely disordered eating behaviors. The percentage of fourth-year students (EAT-26 scores ≥ 20) was 1.3% of males and 0.0% of females before the pandemic and 0.6% of males and 5.8% of females during the pandemic. The percentage of fourth-year students (BITE scores ≥ 20) was 1.3% of males and 2.8% of females before the pandemic and 3.8% of males and 8.0% of females during the pandemic.

**Table 2:** Results of the questionnaires.

	Before the pandemic		During the pandemic		F-Value	*p*-Value
	Mean (SD)		Mean (SD)		(grade × time)	
	First-year	Fourth-year	First-year	Fourth-year		
Male	n = 152		n = 157			
BDI-II	12.3 (0.1)	12.0 (0.6)	12.3 (0.1)	10.8 (0.6)	1.76	0.18
EAT-26	4.0 (0.3)	2.8 (0.3)	3.9 (0.3)	2.9 (0.3)	0.29	0.58
BITE	6.0 (0.3)	5.5 (0.3)	7.2 (0.3)	6.2 (0.3)	0.62	0.43
CISS-T	54.5 (0.8)	52.5 (0.8)	53.3 (0.7)	53.6 (0.8)	3.39	0.06
CISS-E	46.0 (0.7)	43.3 (0.8)	47.4 (0.7)	44.4 (0.8)	0.03	0.84
CISS-A	41.2 (0.8)	40.8 (0.7)	41.5 (0.8)	42.0 (0.7)	0.55	0.45
Female	n = 109		n = 137			
BDI-II	12.4 (0.2)	13.2 (0.9)	12.5 (0.1)	11.5 (0.8)	2.29	0.13
EAT-26	4.2 (0.4)	3.0 (0.5)	5.3 (0.4)	4.5 (0.4)	0.21	0.64
BITE	7.1 (0.6)	7.0 (0.4)	8.0 (0.5)	9.5 (0.4)	4.92	0.02*
CISS-T	51.3 (0.9)	48.8 (0.9)	52.5 (0.8)	52.5 (0.8)	3.29	0.07
CISS-E	45.6 (0.8)	44.8 (0.9)	48.6 (0.7)	45.5 (0.8)	2.70	0.10
CISS-A	40.8 (0.9)	45.3 (0.9)	43.5 (0.8)	46.0 (0.8)	2.95	0.08

SD, standard deviation; n, number of students.

BDI-II, Beck Depression Inventory-II, EAT-26, Eating Attitudes Test-26.

BITE, Bulimic Inventory Test, Edinburgh, CISS, Coping inventory for stressful situations.

*p < 0.05.

We examined the correlation between the BDI-II, EAT, and BITE. Before the pandemic, a significant positive correlation between BDI-II and EAT scores was observed for females in the fourth year (*r* = 0.254, *p* < 0.01). During the pandemic, significant positive correlations were observed in males (*r* = 0.237, *p* < 0.01) and females (*r* = 0.354, *p* < 0.001) in the fourth year. Furthermore, before the pandemic, significant positive correlations between BDI-II and BITE scores were observed for males both at university entrance (*r* = 0.200, *p* < 0.05) and in the fourth year (*r* = 0.240, *p* < 0.01) and for females in the fourth year (*r* = 0.215, *p* < 0.05). During the pandemic, significant positive correlations were observed for males both at university entrance (*r* = 0.165, *p* < 0.05) and in the fourth year (*r* = 0.215, *p* < 0.05) and for females both at university entrance (*r* = 0.187, *p* < 0.05) and in the fourth year (*r* = 0.444, *p* < 0.001).

### Comparison of questionnaires before and during the pandemic

We conducted a two-way ANOVA on the grade and time for each scale to compare scores before and during the pandemic. The results are summarized in **Table 2**. For females, the two-way ANOVA revealed significant interaction effects [BITE: *F*_(1,244)_= 4.92, *p* < 0.05]. Next, we examined the simple main effects of grade and time. During the pandemic, fourth-year students scored significantly higher on the BITE than when they entered university (*p* < 0.01). For males, the two-way ANOVA revealed no significant interaction effects for any scale.

### Three categories of depressive symptoms in the fourth year

The three categories of depressive symptoms in the fourth year before the pandemic were: clinical (19.7% of males, 25.7% of females), subthreshold (38.8% of males, 36.7% of females), and healthy (41.4% of males, 37.6% of females). During the pandemic, the categories were clinical (19.1% of males, 27.7% of females), subthreshold (29.3% of males, 24.8% of females), and healthy (51.6% of males, 47.4% of females). The chi-squared test revealed no significant differences before and during the pandemic, both for males and females. The results are summarized in **Table 3**.

**Table 3:** Three categories of depressive symptoms in the fourth year.

		Before the pandemic	During the pandemic
Male			
Clinical	n (%)	30 (19.7)	30 (19.1)
	asr	0.1	-0.1
Subthreshold	n (%)	59 (38.8)	46 (29.3)
	asr	1.8	-1.8
Healthy	n (%)	63 (41.4)	81 (51.6)
	asr	-1.8	1.8
Female			
Clinical	n (%)	28 (25.7)	38 (27.7)
	asr	-0.4	0.4
Subthreshold	n (%)	40 (36.7)	34 (24.8)
	asr	2.0	-2.0
Healthy	n (%)	41 (37.6)	65 (47.4)
	asr	-1.5	1.5

n, number of students; asr, adjusted standardized residual.

### Comparison of CISS scores among three groups of depression in the fourth year

Before the pandemic, the two-way ANOVA on grade and group for the CISS scores showed significant interaction effects [CISS-E: *F*_(2,149)_ = 6.89, *p* < 0.01] for males. The results are summarized in **Table 4**. Next, we examined the simple main effects of grade and group, and the results differed significantly in grade and group. There were significant differences in the CISS-E scores between the exacerbated and improved groups (*p* < 0.001) and between the exacerbated and unchanged groups (*p* < 0.01) in the fourth year. The CISS-E scores of the improved group was significantly lower in the fourth year than in the first year (*p* < 0.001). For females, the two-way ANOVA revealed significant interaction effects [CISS-E: *F*_(2,106)_ = 7.94, *p* < 0.001]. We examined the simple main effects of the grades and groups. For the CISS-E, there were significant differences between the exacerbated and improved groups (*p* < 0.001) and between the improved and unchanged groups (*p* < 0.01) in the fourth year. The CISS-E scores of the exacerbated group were significantly higher (*p* < 0.05), and the scores of the improved group were significantly lower (*p* < 0.01) in the fourth year than in the first year.

**Table 4:** Comparison of CISS scores among three groups in the fourth year before the pandemic.

	Exacerbated group		Unchanged group		Improved group		F-Value	*p*-Value
	Mean (SD)		Mean (SD)		Mean (SD)		(grade × group)	
	First-year	Fourth-year	First-year	Fourth-year	First-year	Fourth-year		
Male	n = 30		n = 59		n = 63			
CISS-T	51.7 (1.7)	50.2 (1.7)	54.9 (1.2)	50.6 (1.2)	55.2 (1.1)	55.3 (1.1)	2.59	0.07
CISS-E	48.5 (1.6)	50.2 (1.8)	44.6 (1.1)	43.2 (1.2)	46.0 (1.1)	39.9 (1.2)	6.89	0.001^**^
CISS-A	43.0 (1.8)	42.2 (1.7)	40.6 (1.3)	38.2 (1.2)	40.8 (1.2)	42.3 (1.1)	2.26	0.1
Female	n = 28		n = 40		n = 41			
CISS-T	52.1 (1.9)	46.2 (1.9)	50.8 (1.6)	47.8 (1.6)	51.4 (1.6)	51.7 (1.6)	2.71	0.07
CISS-E	46.0 (1.6)	50.2 (1.7)	45.7 (1.4)	46.0 (1.4)	45.3 (1.3)	40.1 (1.4)	7.94	<0.001^***^
CISS-A	40.0 (1.7)	42.3 (1.5)	41.0 (1.4)	44.9 (1.3)	41.1 (1.4)	47.7 (1.3)	1.91	0.15

SD, standard deviation; n, number of students; CISS, Coping inventory for stressful situations.

**p < 0.01, ***p < 0.001.

During the pandemic, the two-way ANOVA on grade and group for the CISS scores showed significant interaction effects [CISS-T: *F*_(2,154)_ = 6.93, *p* < 0.01; CISS-E: *F*_(2,154)_ = 3.70, *p* < 0.05] for males. The results are summarized in **Table 5**. We examined the simple main effects of the grades and groups. For the CISS-T scores, there was a significant difference between the exacerbated and improved groups in the fourth year (*p* < 0.05). The CISS-T scores of the exacerbated group were significantly lower (*p* < 0.01), and the score of the improved group was significantly higher (*p* < 0.05) in the fourth year than in the first year. For the CISS-E scores, significant differences were observed between the improved group and the exacerbated and unchanged groups in the fourth year (*p* < 0.05). The CISS-E scores of the improved group were significantly lower in the fourth year than in the first year (*p* < 0.001). For females, two-way ANOVA on grade and group for the CISS scores showed significant interaction effects [CISS-E: *F*_(2,134)_ = 9.86, *p* < 0.001]. We examined the simple main effects of grade and group, and the results differed significantly in grade and group. There were significant differences in the CISS-E scores between the exacerbated and improved groups (*p* < 0.001) and between the improved and unchanged groups (*p* < 0.05) in the fourth year. The CISS-E scores of the improved group were significantly lower in the fourth year than in the first year (*p* < 0.001).

**Table 5:** Comparison of CISS scores among three groups in the fourth year during the pandemic.

	Exacerbated group		Unchanged group		Improved group		*F*	*p*-Value
	Mean (SD)		Mean (SD)		Mean (SD)		(grade × group)	
	First-year	Fourth-year	First-year	Fourth-year	First-year	Fourth-year		
Male	n = 30		n = 46		n = 81			
CISS-T	55.4 (1.9)	49.5 (2.0)	53.5 (1.5)	53.1 (1.6)	52.4 (1.1)	55.4 (1.2)	6.93	0.001**
CISS-E	50.0 (1.7)	49.6 (1.8)	46.5 (1.4)	46.1 (1.5)	46.9 (1.0)	41.5 (1.1)	3.70	0.02^*^
CISS-A	40.8 (2.0)	40.3 (1.7)	41.6 (1.6)	42.0 (1.4)	41.7 (1.2)	42.6 (1.0)	0.18	0.83
Female	n = 38		n = 34		n = 65			
CISS-T	51.2 (1.6)	51.0 (1.6)	52.5 (1.7)	49.6 (1.7)	53.4 (1.2)	54.9 (1.2)	2.06	0.13
CISS-E	50.0 (1.4)	52.8 (1.4)	47.7 (1.5)	45.6 (1.5)	48.3 (1.1)	41.3 (1.1)	9.86	<0.001***
CISS-A	42.2 (1.7)	46.0 (1.7)	40.7 (10.0)	44.2 (8.1)	43.7 (10.4)	47.5 (9.9)	0.79	0.45

SD, standard deviation, n, number of students, CISS, Coping inventory for stressful situations.

*p < 0.05, **p < 0.01,***p < 0.001.

## Discussion

In this study, there was no significant difference in the frequency of depressive symptoms for students in the fourth year before and during the pandemic; however, female students had an increase in bulimic symptoms during the pandemic. Additionally, although the decrease in CISS-E scores was commonly associated with a reduction in depressive symptoms both before and during the pandemic, the decrease in CISS-T scores was associated with an increased risk of depressive symptoms only in males during the pandemic.

For depressive symptoms, there was no significant interaction effect on BDI-II between grade and time for males and females. Contrary to our prediction, no significant difference was observed in the frequency of depressive symptoms before and during the pandemic. According to a recent review, a substantial group of individuals has been largely unaffected or are even performed better during the pandemic (48). A study in the United States reported that adolescents with elevated mental health problems before the pandemic experienced a significant reduction in these problems 1 month after school closures (49). People with depressive symptoms had a detrimental impact on their mental health during the pandemic, although did not seem to have further increased symptom severity compared with their pre-pandemic levels (50). The results of this study are similar to those of previous studies. There are several possible explanations for this. Staying at home due to the pandemic gave students fewer opportunities for interpersonal communication and helped them build a structured and fixed daily routine, which has been expressed as a preferable setting for providing a feeling of safety (51). A study of adolescents reported that almost half the participants felt that the pandemic also exerted positive effects (52). However, social isolation and depression are likely to interact with each other (53), and distancing and social isolation were adopted around the world during the pandemic. A previous study suggested that young adults had the potential to experience resilience during the pandemic, had a relatively low risk of health complications from COVID-19, and were competent at using social media to connect with others (54). Social media is a representative tool that enables communication and social exchange with others even when isolated, especially for younger people (55). Although the pandemic caused unusual environmental stress in university students, some students might also have experienced a preferable impact on their new lifestyle, thus there might be no significant difference in the frequencies of depressive symptoms in the fourth year before and during the pandemic. However, we did not examine how students felt about the changes in their environment during the pandemic. Therefore, further research is needed.

The BITE scores were significantly higher for fourth-year female students during the pandemic than when they entered university. There was a significant interaction effect between grade and time on BITE scores for females. Positive correlations were observed between BDI-II and BITE scores for males and females in the fourth year, and the percentage of fourth-year students with severely disordered eating behaviors increased during the pandemic compared to before the pandemic. Several studies on eating disorders have reported worsening symptoms during the pandemic (28, 29). Women’s body dissatisfaction and physical appearance comparisons seem to have increased throughout the pandemic (56). Recent studies have determined that adolescents still suffer from problematic eating (mostly binge eating) that began during the pandemic and express concerns about their appearance (57). A study on college students reported that frequencies of bulimia nervosa or binge eating disorders were greater during (vs. before) the pandemic, which was not observed for anorexia nervosa (58). These findings are consistent with our results. In stressful and fearful situations, such as during a pandemic, changes in everyday eating behaviors may occur (59). Touyz et al. (60) suggested that higher rates of bulimia nervosa or binge eating disorders might be explained by stockpiling groceries, restrictions in exercising, and difficulty in abstaining from food while at home (58, 60). The problematic eating behaviors persisted once they appeared. The presence of any degree of eating disorder symptoms is associated with decreased quality of life compared to individuals without any symptoms (61). Several studies have reported that bulimic symptoms are highly comorbid with depressive symptoms and associated with the development of depressive symptoms (62). Thus, the early detection of bulimic symptoms in university students is necessary. Our results suggest that it is important to pay attention not only to depressive symptoms but also to bulimic symptoms during the pandemic.

Stress coping behaviors affect the occurrence of stress−related diseases (26, 27). The strategies that may be used to cope with stress and/or trauma will differ between individuals (63, 64). A recent study of academic staff of a university reported that the coping strategy utilized by the respondents was taking alcohol or drugs and few of the respondents used a positive reinterpretation and growth as a coping style as they looked for something good in what is happening (65). A study of firefighters showed that firefighters using the coping mechanisms of minimization and blame were associated with the greater likelihood of PTSD (64). Previous studies on adolescents suggested that stress coping style is a factor contributing to their increased vulnerability to depression (25). In this study, there was a significant interaction effect between the grade and depression groups on the CISS-E scores of males and females both before and during the pandemic. Emotion-oriented coping is an emotional response to a problem that is considered a non-adaptive aspect of coping and is associated with psychological distress (66). Reducing non-adaptive coping behaviors may have the most positive impact on reducing depression, anxiety, and stress (67). Similar to previous reports, this study showed that a decrease in CISS-E scores in the depression-improved group was common in males and females before and during the pandemic. Additionally, a significant interaction effect was observed between grade and depression group on CISS-T scores of males during the pandemic, which was not observed before the pandemic in this study. Compared to the improved group, males in the exacerbated group had significantly lower CISS-T scores in their fourth year in males. Task-oriented coping involves solving a problem, cognitive restructuring of the problem, or attempts to alter the situation, and aims to directly manage a stressor to reduce distress. A review of methods used by the general population to cope with emergent infectious diseases reported that task-oriented coping allowed respondents to actively reduce feelings of uncertainty and increase feelings of control over their health in the 24 studies analyzed (68, 69). A previous study suggested that psychological support focused on strengthening adaptive strategies of coping with stressful situations is important (12). One study showed that task-oriented coping was an effective approach to reducing stress during the pandemic (24, 70). Coping is effective when an individual can moderate stress (45). The controllability of a situation can be either real or perceived (68, 71). Male students who tend to use task-oriented coping may perceive unusual environmental stresses as controllable and reduce depressive symptoms. A recent study suggested that the awareness of an effective and adaptive coping strategy is imperative to safeguard the mental health (65). Therefore, in stressful situations such as the pandemic, this result suggests that increasing task-oriented coping might be associated with reduced depressive symptoms, especially in males.

This study has a few limitations. First, this study only considered depressive symptoms, eating behaviors, and stress coping as effects of the pandemic. Future studies should consider how students feel about the changes in their environments during the pandemic. Second, depressive symptoms or eating behaviors can be affected by socio-economic status, other mental health conditions, cultural factors and many other factors. Future studies should address these factors. Third, our results were limited to a single university, fourth-year students, and a certain period. Future research should be expanded to other years and include other universities. Finally, we did not conduct interim assessments in this study. Longitudinal data collection could provide more robust insights. Future studies should include interim assessments.

## Conclusions

Our results showed that the COVID-19 pandemic did not increase the rate of depressive symptoms among fourth-year university students who had subthreshold depression upon university entrance. However, there was a difference in changes for stress-coping behaviors associated with an increased risk of depressive symptoms. Additionally, the pandemic increased bulimic symptoms of female students. Problematic eating behaviors persist once they appear and are associated with the development of depressive symptoms. Therefore, we should monitor not only depressive symptoms but also bulimic symptoms and stress-coping behaviors among university students with subthreshold depression to prevent deterioration from subthreshold depression to clinical depression during s pandemic. The results of this study may contribute evidence to provide future psychological support and early intervention for stressful situations among university students.

## Data Availability

The original contributions presented in the study are included in the article/supplementary material. Further inquiries can be directed to the corresponding author.

## References

[1] Shen C Smith RB Heller J Spiers ADV Thompson R Ward H. Depression and anxiety in adolescents during the COVID-19 pandemic in relation to the use of digital technologies: longitudinal cohort study. J Med Internet Res. (2024) 7:26:e45114. doi: 10.2196/45114 PMC10882466 38324379

[2] Nagib N Horita R Miwa T Adachi M Tajirika S Imamura N. Impact of COVID-19 on the mental health of Japanese university students (years II-IV). Psychiatry Res. (2023) 325:115244. doi: 10.1016/j.psychres.2023.11524 37182282

[3] Li Y Wang A Wu Y Han N Huang H. Impact of the COVID-19 pandemic on the mental health of college students: a systematic review and meta analysis. Front Psychol. (2021) 12:669119. doi: 10.3389/fpsyg.2021.669119 34335381 PMC8316976

[4] Fruehwirth JC Biswas S Perreira KM. The Covid-19 pandemic and mental health of first-year college students: examining the effect of Covid-19 stressors using longitudinal data. PloS One. (2021) 16:e0247999. doi: 10.1371/journal.pone.0247999 33667243 PMC7935268

[5] Wang C Wen W Zhang H Ni J Jiang J Cheng Y. Anxiety, depression, and stress prevalence among college students during the COVID-19 pandemic: a systematic review and meta-analysis. J Am Coll Health. (2023) 71:2123–30. doi: 10.1080/07448481.2021.1960849 34469261

[6] Son C Hegde S Smith A Wang X Sasangohar F. Effects of COVID-19 on college students’ mental health in the United States: Interview survey study. J Med Internet Res. (2020) 22:e21279. doi: 10.2196/21279 32805704 PMC7473764

[7] Takagaki K Yokoyama S. Factors associated with university students’ deterioration from subthreshold depression to depression before and during the COVID-19 pandemic. Behav Sci (Basel). (2023) 13:72. doi: 10.3390/bs13010072 36661644 PMC9854505

[8] Fuse-Nagase Y Marutani T Tachikawa H Iwami T Yamamoto Y Moriyama T. Increase in suicide rates among undergraduate students in Japanese national universities during the COVID-19 pandemic. Psychiatry Clin Neurosci. (2021) 75:351–2. doi: 10.1111/pcn.13293 PMC8446983 34355847

[9] Horita R Nishio A Yamamoto M. Lingering effects of COVID-19 on the mental health of first-year university students in Japan. PloS One. (2022) 17:e0262550. doi: 10.1371/journal.pone.0262550 35020752 PMC8754334

[10] Nagasu M Muto K Yamamoto I. Impacts of anxiety and socioeconomic factors on mental health in the early phases of the COVID-19 pandemic in the general population in Japan: A web-based survey. PloS One. (2021) 16:e0247705. doi: 10.1371/journal.pone.0247705 33730044 PMC7968643

[11] Raval N. Mental health implications of COVID-19 in India. IJHW. (2020) 11:276–81.

[12] Twardowska-Staszek E Biel K Rostek I Seredyńska A. Causes of stress among poles and how they cope with stress during the COVID-19 pandemic. Front Psychiatry. (2022) 6:829918. doi: 10.3389/fpsyt.2022.829918 PMC9020827 35463496

[13] Pincus HA Davis WW McQueen LE. Subthreshold mental disorders. A review and synthesis of studies on minor depression and other brand names. Br J Psychiatry. (1999) 174:288–96. doi: 10.1192/bjp.174.4.288 10533546

[14] Noyes BK Munoz DP Khalid-Khan S Brietzke E Booij L. Is subthreshold depression in adolescence clinically relevant? J Affect Disord. (2022) 309:123–30. doi: 10.1016/j.jad.2020.03.111 35429521

[15] Lee YY Stockings EA Harris MG Doi SAR Page IS Davidson SK. The risk of developing major depression among individuals with subthreshold depression: A systematic review and meta-analysis of longitudinal cohort studies. Psychol Med. (2019) 49:92–102. doi: 10.1017/S0033291718000557 29530112

[16] Balázs J Miklósi M Keresztény A Hoven CW Carli V Wasserman C. Adolescent subthreshold-depression and anxiety: Psychopathology, functional impairment, and increased suicide risk. J Child Psychol Psychiatry. (2013) 54:670–7. doi: 10.1111/jcpp.12016 23330982

[17] Shiraishi N Sakata M Toyomoto R Yoshida K Luo Y Nakagami Y. Three types of university students with subthreshold depression characterized by distinctive cognitive behavioral skills. Cognit Behav Ther. (2024) 53:207–19. doi: 10.1080/16506073.2023.2288557 38008940

[18] Torales J Rios-Gonzalez C Barrios L O’Higgins M González I Garcia O. Selfperceived stress during the quarantine of the COVID-19 pandemic in Paraguay: an exploratory survey. Front Psychiatry. (2020) 11:558691. doi: 10.3389/fpsyt.2020.558691 33192674 PMC7649175

[19] Langer ÁI Crockett MA Bravo-Contreras M Carrillo-Naipayan C Chaura-Marió M Gómez-Curumilla B. Social and economic factors associated with subthreshold and major depressive episode in university students during the COVID-19 pandemic. Front Public Health. (2022) 18:893483. doi: 10.3389/fpubh.2022.893483 PMC9157787 35664111

[20] Johnson JG Cohen P Kasen S. Minor depression during adolescence and mental healthoutcomes during adulthood. Br J Psychiatry. (2009) 195:264–5. doi: 10.1192/bjp.bp.108.054239 PMC2801821 19721119

[21] Strzelecka A Gotlib-Małkowska J Chmielewski J Wojciechowska M Kordyzon M Rębak D. Effect of COVID-19 pandemic on health behaviours of students of medical specialities from the aspect of psychological support based on a Polish example. Ann Agric Environ Med. (2024) 31:560–5. doi: 10.26444/aaem/193622 39743715

[22] Güzel Â Mutlu NL Molendijk M. COVID-19-related changes in eating disorder pathology, emotional and binge eating and need for care: a systematic review with frequentist and Bayesian meta-analyses. Eat Weight Disord. (2023) 28:19. doi: 10.1007/s40519-023-01547-2 36805344 PMC9941242

[23] Haghshomar M Shobeiri P Brand S Rossell SL Akhavan Malayeri A Rezaei N. Changes of symptoms of eating disorders (ED) and their related psychological health issues during the COVID-19 pandemic: a systematic review and meta-analysis. J Eat Disord. (2022) 10:1–8. doi: 10.1186/s40337-022-00550-9 35418108 PMC9006500

[24] Götmann A Bechtoldt MN. Coping with COVID-19 - Longitudinal analysis of coping strategies and the role of trait mindfulness in mental well-being. Pers Individ Dif. (2021) 175:110695. doi: 10.1016/j.paid.2021.110695 33531724 PMC7843110

[25] Nolen-Hoeksema S Girgus JS. The emergence of gender differences in depression during adolescence. Psychol Bull. (1994) 115:424–43. doi: 10.1037/0033-2909.115.3.424 8016286

[26] Javadi-Pashaki N Darvishpour A. Survey of stress and coping strategies to predict the general health of nursing staff. J Educ Health Promot. (2019) 8:74. doi: 10.4103/jehp.jehp_355_18 31143791 PMC6512229

[27] Zeighami M Pour Bahaadini Zarandi N. The relationship between academic achievement and students’ general health and coping styles: A study on nursing, midwifery and health students of Islamic Azad University – Kerman branch. Strides Dev Med Educ. (2011) 8:41−8.

[28] Branley-Bell D Talbot CV. Exploring the impact of the COVID-19 pandemic and UK lockdown on individuals with experience of eating disorders. J Eat Disord. (2020) 8:44. doi: 10.1186/s40337-020-00319-y 32874585 PMC7444862

[29] Schlegl S Maier J Meule A Voderholzer U. Eating disorders in times of the COVID-19 pandemic-results from an online survey of patients with anorexia nervosa. Int J Eat Disord. (2020) 53:1791–800. doi: 10.1002/eat.23374 PMC7461418 32841413

[30] De Pasquale C Sciacca F Conti D Pistorio ML Hichy Z Cardullo RL. Relations between mood states and eating behavior during COVID-19 pandemic in a sample of italian college students. Front Psychol. (2021) 12:684195. doi: 10.3389/fpsyg.2021.684195 34367004 PMC8333995

[31] Jinnin R Okamoto Y Takagaki K Nishiyama Y Yamamura T Okamoto Y. Detailed course of depressive symptoms and risk for developing depression in late adolescents with subthreshold depression: a cohort study. Neuropsychiatr Dis Treat. (2016) 13:25–33. doi: 10.2147/NDT.S117846 28053534 PMC5191576

[32] Miyake Y Okamoto Y Takagaki K Yoshihara M. Changes in eating attitudes and risk for developing disordered eating behaviors in college students with subthreshold eating disorders: A cohort study. Psychopathology. (2023) 56:276–84. doi: 10.1159/000527604 36509080

[33] Beck A Steer R Brown G. Manual for the beck depression inventory-II. San Antonio, TX: Psychological Corporation (1996).

[34] Garner DM Olmsted MP Bohr Y Garfinkel PE. The eating attitudes test: psychometric features and clinical correlates. Psychol Med. (1982) 12:871–8. doi: 10.1017/s0033291700049163 6961471

[35] Henderson M Freeman CP. A self-rating scale for bulimia. The ‘BITE’. Br J Psychiatry. (1987) 150:18–24. doi: 10.1192/bjp.150.1.18 3651670

[36] Endler NS Parker JDA. Coping inventory for stressful situations (CISS): manual. Toronto: Malti- Health Systems Inc (1990).

[37] Arnau RC Meagher MW Norris MP Bramson R. Psychometric evaluation of the Beck Depression Inventory-II with primary care medical patients. Health Psychol. (2001) 20:112–9. doi: 10.1037//0278-6133.20.2.112 11315728

[38] Kojima M Furukawa T. Japanese version of the beck depression inventory. 2nd ed. Tokyo: Nippon-Hyoron-sha Co, Tokyo (2003).

[39] Kojima M Furukawa TA Takahashi H Kawai M Nagaya T Tokudome S. Cross-cultural validation of the beck depression inventory-II in Japan. Psychiatry Res. (2002) 110:291–9. doi: 10.1016/s0165-1781(02)00106-3 12127479

[40] Mann AH Wakeling A Wood K Monck E Dobbs R Szmukler G. Screening for abnormal eating attitudes and psychiatric morbidity in an unselected population of 15-year-old schoolgirls. Psychol Med. (1983) 13:573–80. doi: 10.1017/s0033291700047991 6622610

[41] Siervo M Boschi V Papa A Bellini O Falconi C. Application of the SCOFF, eating attitudes test 26 (EAT 26) and eating inventory (TFEQ) questionnaires in young women seeking diet-therapy. Eat Weight Disord. (2005) 10:76–82. doi: 10.1007/BF03327528 16114220

[42] Nakai Y. Validity of the Japanese version of eating attitudes test (EAT). Seishin Igaku. (2003) 45:161–5.

[43] Mukai T Crago M Shisslak CM. Eating attitudes and weight preoccupation among female high school students in Japan. J Child Psychol Psychiatry. (1994) 35:677–88. doi: 10.1111/j.1469-7610.1994.tb01213.x 8040220

[44] Nakai Y Noma S. Evaluation method of eating disorder symptoms. Modern Physician. (2007) 27:785–8.

[45] Jensen C Forlini C Partridge B Hall W. Australian university students’ coping strategies and use of pharmaceutical stimulants as cognitive enhancers. Front Psychol. (2016) 7:277. doi: 10.3389/fpsyg.2016.00277 26973573 PMC4771940

[46] Furukawa T Suzuki-Moor A Saito Y Hamanaka T. Reliability and validity of the Japanese version of the coping inventory for stressful situations (CISS): a contribution to the cross-cultural studies of coping. Seishin Shinkeigaku Zasshi. (1993) 95:602–20. doi: 10.2466/08.02.PR0.116k23w6 8234537

[47] Watanabe K Yokoyama K Furukawa TA. Reliability and validity of the Japanese version of the coping inventory for adults for stressful situations in healthy people. Psychol Rep. (2015) 116:447–69. doi: 10.2466/08.02.PR0.116k23w6 25826435

[48] Manchia M Gathier AW Yapici-Eser H Schmidt MV de Quervain D van Amelsvoort T. The impact of the prolonged COVID-19 pandemic on stress resilience and mental health: A critical review across waves. Eur Neuropsychopharmacol. (2022) 55:22–83. doi: 10.1016/j.euroneuro.2021.10.864 34818601 PMC8554139

[49] Penner F Hernandez OJ Sharp C. Change in youth mental health during the COVID-19 pandemic in a majority Hispanic/Latinx US sample. J Am Acad Child Adolesc Psychiatry. (2021) 60:513–23. doi: 10.1016/j.jaac.2020.12.027 33359408

[50] Pan KY Kok AAL Eikelenboom M Horsfall M Jorg F Luteijn RA. The mental health impact of the COVID-19 pandemic on people with and without depressive, anxiety, or obsessive-compulsive disorders: a longitudinal study of three Dutch case-control cohorts. Lancet Psychiatry. (2021) 8:121–9. doi: 10.1016/S2215-0366(20)30491-0 PMC7831806 33306975

[51] Pelto-Piri V Wallsten T Hylen U Nikban I Kjellin L. Feeling safe or unsafe in psychiatric inpatient care, a hospital-based qualitative interview study with inpatients in Sweden. Int J Ment Health Syst. (2019) 13:23–32. doi: 10.1186/s13033-019-0282-y 30996733 PMC6452515

[52] Oosterhoff B Palmer CA Wilson J Shook N. Adolescents’ Motivations to engage in social distancing during the COVID-19 pandemic: associations with mental and social health. J Adolesc Health. (2020) 67:179–85. doi: 10.1016/j.jadohealth.2020.05.004 PMC7205689 32487491

[53] Zhu S Kong X Han F Tian H Sun S Sun Y. Association between social isolation and depression: Evidence from longitudinal and Mendelian randomization analyses. J Affect Disord. (2024) 350:182–7. doi: 10.1016/j.jad.2024.01.106 38220103

[54] Shanahan L Steinhoff A Bechtiger L Murray AL Nivette A Hepp U. Emotional distress in young adults during the COVID-19 pandemic: evidence of risk and resilience from a longitudinal cohort study. Psychol Med. (2022) 52:824–33. doi: 10.1017/S003329172000241X PMC7338432 32571438

[55] Jo H. Effects of psychological discomfort on social networking site (SNS) usage intensity during COVID-19. Front Psychol. (2022) 13:939726. doi: 10.3389/fpsyg.2022.939726 35936310 PMC9354781

[56] Vall-Roqué H Andrés A González-Pacheco H Saldaña C. Women’s body dissatisfaction, physical appearance comparisons, and Instagram use throughout the COVID-19 pandemic: A longitudinal study. Int J Eat Disord. (2023) 56:118–31. doi: 10.1002/eat.23827 PMC10092427 36268646

[57] Kim S Aum T Lee DG. Depression in the COVID-19 endemic era: Analysis of online self- disclosures by young South Koreans. PloS One. (2024) 19:e0314881. doi: 10.1371/journal.pone.0314881 39724057 PMC11671000

[58] Kim H Rackoff GN Fitzsimmons-Craft EE Shin KE Zainal NH Schwob JT. College mental health before and during the COVID-19 pandemic: results from a nationwide survey. Cognit Ther Res. (2022) 46:1–10. doi: 10.1007/s10608-021-10241-5 PMC8214371 34177004

[59] Haddad C Zakhour M Kheir MB Haddad R Hachach MA Sacre H. Associationbetween eating behavior and quarantine/confinement stressors during the coronavirus disease 2019 outbreak. J Eat Disord. (2020) 8:40. doi: 10.1186/s40337-020-00317-0 32879730 PMC7458649

[60] Touyz S Lacey H Hay P. Eating disorders in the time of COVID-19. J Eat Disord. (2020) 8:19. doi: 10.1186/s40337-020-00295-3 32337045 PMC7170399

[61] Mond JM Myers TC Crosby RD Hay PJ Mitchell JE. Bulimic eating disorders in primary care: Hidden morbidity still? J Clin Psychol Med Settings. (2010) 17:56–63. doi: 10.1007/s10880-009-9180-9 20039194

[62] Crow SJ Peterson CB Swanson SA Raymond NC Specker S Eckert ED. Increased mortality in bulimia nervosa and other eating disorders. Am J Psychiaty. (2009) 166:1342–6. doi: 10.1176/appi.ajp.2009.09020247 19833789

[63] Nydegger R Nydegger L Basile F. Post-traumatic stress disorder and coping among career professional firefighters. Am J Health Sci. (2011) 2:11–20. doi: 10.19030/ajhs.v2i1.4365

[64] Theleritis C Psarros C Mantonakis L Roukas D Papaioannou A Paparrigopoulos T. Coping and its relation to PTSD in greek firefighters. J Nerv Ment Dis. (2020) 208:252–9. doi: 10.1097/NMD.0000000000001103 31913955

[65] Owoeye I Agunbiade T Agboola A Sanya O Adebiyi B Akimanimpaye F. Health SA. Assessing the psychological distress and coping strategies among academic staff of a university during COVID-19. (2025) 30:2752. doi: 10.4102/hsag.v30i0.2752 PMC11966654 40183029

[66] Spoor ST Bekker MH van Strien T van Heck GL. Relations between negative affect, coping, and emotional eating. Appetite. (2007) 48:368–76. doi: 10.1016/j.appet.2006.10.005 17145096

[67] Mahmoud JS Staten R Hall LA Lennie TA. The relationship among young adult college students’ depression, anxiety, stress, demographics, life satisfaction, and coping styles. Issues Ment Health Nurs. (2012) 33:149–56. doi: 10.3109/01612840.2011.632708 22364426

[68] Savary A Hammouda M Genet L Godet C Bunel V Weisenburger G. Coping strategies, anxiety and depression related to the COVID-19 pandemic in lung transplant candidates and recipients. Results from a monocenter series. Respir Med Res. (2021) 80:100847. doi: 10.1016/j.resmer.2021.100847 34371237 PMC8260501

[69] Chew Q Wei K Vasoo S Chua HC Sim K. Narrative synthesis of psychological and coping responses towards emerging infectious disease outbreaks in the general population: practical considerations for the COVID-19 pandemic. . Singapore Med J. (2020) 61:350–6. doi: 10.11622/smedj.2020046 PMC7926608 32241071

[70] Rossi MF Gualano MR Magnavita N Moscato U Santoro PE Borrelli I. Coping with burnout and the impact of the COVID-19 pandemic on workers’ mental health: A systematic review. Front Psychiatry. (2023) 14:1139260. doi: 10.3389/fpsyt.2023.1139260 37009102 PMC10060559

[71] Bruchon-Schweitzer M. Coping and adjustment strategies for dealing with stress. Rech Soins Infirm. (2001) 67:68–83. 21374910

